# Change in Default Prescription Length and Statin Prescribing Behavior

**DOI:** 10.1001/jamainternmed.2025.0185

**Published:** 2025-04-07

**Authors:** Mili Mehta, Alexander C. Fanaroff, Corinne M. Rhodes, Aria Xiong, Christopher K. Snider, E. Madeline Grenader, Michael O. Harhay, Nune Mehrabyan, Maryanne K. Peifer, Kevin G. M. Volpp, M. Kit Delgado

**Affiliations:** 1 Division of Cardiology, Department of Medicine, Tufts University School of Medicine, Boston, Massachusetts; 2 Division of Cardiovascular Medicine, Department of Medicine, Perelman School of Medicine, University of Pennsylvania, Philadelphia; 3 Center for Health Incentives and Behavioral Economics, University of Pennsylvania, Philadelphia; 4 Division of General Internal Medicine, Department of Medicine, Perelman School of Medicine, University of Pennsylvania, Philadelphia; 5 Penn Medicine Nudge Unit, Center for Health Care Transformation and Innovation, University of Pennsylvania Health System, Philadelphia; 6 Department of Biostatistics, Epidemiology, and Informatics, Perelman School of Medicine, University of Pennsylvania, Philadelphia; 7 Information Services, University of Pennsylvania Health System, Philadelphia; 8 Department of Family Medicine and Community Health, Perelman School of Medicine, University of Pennsylvania, Philadelphia; 9 Department of Medical Ethics and Health Policy, Perelman School of Medicine, University of Pennsylvania, Philadelphia; 10 Department of Emergency Medicine, Perelman School of Medicine, University of Pennsylvania, Philadelphia

## Abstract

This quality improvement study examines the association of changing the default supply for statin prescriptions to 90 days with clinician prescribing behavior.

Statins are cost-effective, evidence-based medications that reduce the incidence, morbidity, and mortality of atherosclerotic cardiovascular disease,^1^ yet many patients are not adherent.^2,3^ Having medication available is a necessary precondition for adherence, and longer prescription durations facilitate adherence by reducing the frequency with which patients must act to obtain medications.^4,5^ However, many patients are not prescribed 90-day supplies of statins. Higher rates of medication possession for statins are associated with lower all-cause mortality,^6^ highlighting the potential clinical benefits of longer prescription duration. In this study, we sought to examine the association of changing the default supply for statin prescriptions to 90 days with clinician prescribing behavior.

## Methods

In the intervention, we set the default prescription length for all statins on the primary care electronic health record (EHR) preference to 90 days in November 2022 across a large academic health system. We compared the proportions of statin prescriptions written for 90 days before and after the default change to levothyroxine, a commonly prescribed medication that did not undergo intervention, using a difference-in-differences analysis to control for natural trends. Results were stratified by race and ethnicity (as self-reported in the EHR), insurance, and median zip code household income. We also assessed the proportion of patients who had a change to their statin prescription within 90 days after the prescription was written before and after the intervention.

This project was reviewed and determined to qualify as quality improvement by the University of Pennsylvania’s institutional review board. We followed SQUIRE reporting guidelines.

## Results

**Figure 1.**
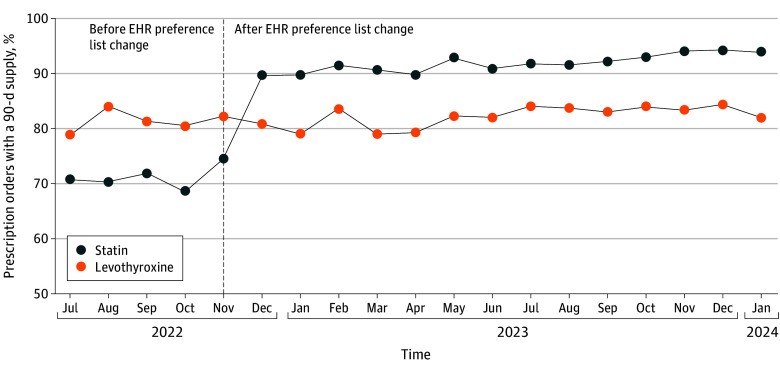
Proportion of Prescription Orders With a 90-Day Supply Before and After Electronic Health Record (EHR) Preference List Default Change

There were 5698 statin prescriptions written prior to the default change (July to November 2022) and 18 530 prescriptions written after (November 2022 to January 2024). The proportion of prescriptions written for a 90-day supply increased from 70.7% to 91.7% following intervention, an adjusted increase of 20.3 percentage points (95% CI, 18.3-22.2 percentage points; *P* < .001) compared to levothyroxine (Figure 1).

**Figure 2.**
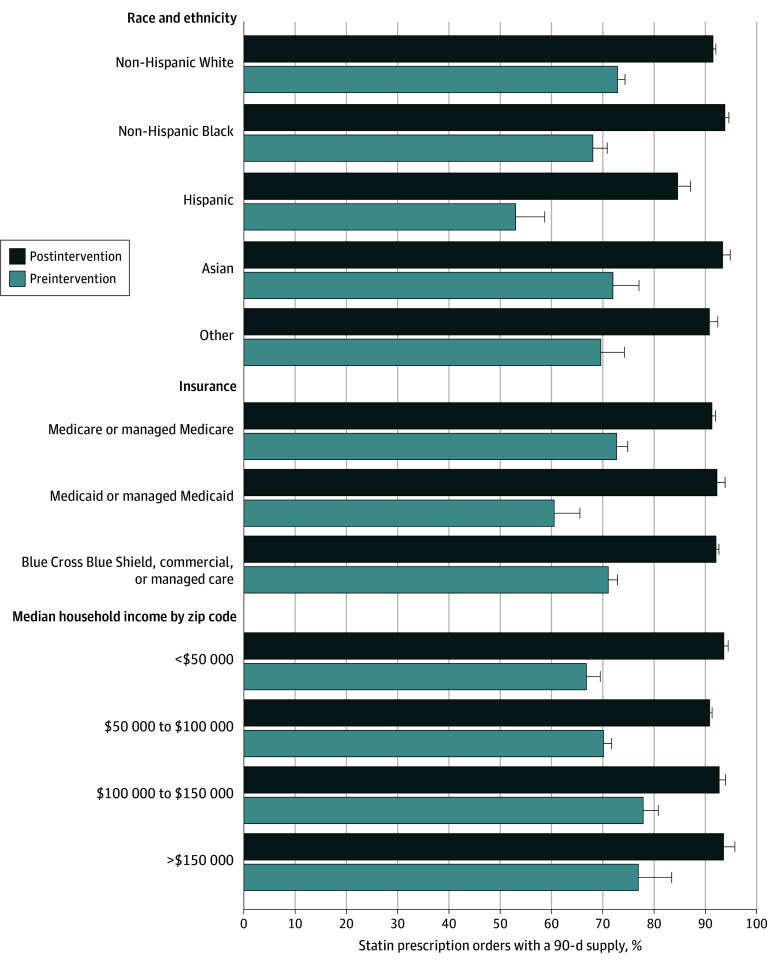
Proportion of Statin Prescription Orders With a 90-Day Supply Before and After Electronic Health Record Preference List Default Change by Patient Race and Ethnicity, Insurance, and Income The other race category includes American Indian or Alaska Native, East Indian, Native Hawaiian or Other Pacific Islander, and unknown, other race, or patient declined to answer; these categories were grouped together owing to small sample sizes. Error bars indicate 95% CIs.

Prior to the intervention, Hispanic and non-Hispanic Black patients, those with Medicaid, and those living in zip codes with median household income lower than $50 000 were less likely to receive 90-day prescriptions (Figure 2). After the default change, all subgroups were equally likely to receive 90-day prescriptions except for Hispanic patients, for whom the gap relative to non-Hispanic White patients was reduced from 19.9 to 6.9 percentage points. Following the intervention, there was no difference in the proportion of patients who had a change in type or strength of statin prescription during the 90-day prescription time period (2.8% vs 2.6%; *P* = .25).

## Discussion

Changing the default statin prescription duration was associated with a 20.3−percentage point increase in proportion of statin prescriptions written for a 90-day supply, while racial and socioeconomic disparities in prescription length were reduced. These findings illustrate that a simple restructuring of choice architecture in the EHR may increase the adoption of evidence-based strategies for improving adherence, without restricting patient or physician choice.

Importantly, baseline rates of 90-day statin prescriptions were notably lower among patients with Medicaid, median household income lower than $50 000, and Hispanic ethnicity and Black race. With the default change, approximately 90% of patients in all groups were prescribed 90-day supplies, and groups with lower baseline rates of 90-day prescriptions had larger effects. Disparities in prescription length prior to the default change may have been mediated by historical policy restrictions on prescription size by some payers, legacy effects from larger co-payments before availability of generic high-potency statins, or clinicians’ implicit bias related to beliefs about patients’ ability to afford larger medication supplies. Regardless of cause, the elimination of disparities after the change in default prescription length highlights how well-designed default changes can reduce health disparities. Moreover, 8% of patient-clinician dyads actively choosing a 30-day supply, presumably for relevant clinical or patient-preference reasons, highlights the freedom of choice preserved by this intervention structure.

Although this study did not assess adherence as a result of the increase in 90-day prescription rates, other studies have demonstrated that 90-day prescription fills are associated with greater adherence^4,5^ and reduced mortality.^6^ Many interventions that attempt to increase adherence are costly, time-intensive, and not sustainable. By contrast, changing default prescription length may sustainably increase adherence without substantial resource investment.

## Notes

Supplement. Data Sharing Statement

## References

[1] Thavendiranathan P, Bagai A, Brookhart MA, Choudhry NK. Primary prevention of cardiovascular diseases with statin therapy: a meta-analysis of randomized controlled trials. Arch Intern Med. 2006;166(21):2307-2313. doi:10.1001/archinte.166.21.2307 17130382

[2] Neiman AB, Ruppar T, Ho M, . CDC grand rounds: improving medication adherence for chronic disease management—innovations and opportunities. MMWR Morb Mortal Wkly Rep. 2017;66(45):1248-1251. doi:10.15585/mmwr.mm6645a2 29145353 PMC5726246

[3] Chan AHY, Cooper V, Lycett H, Horne R. Practical barriers to medication adherence: what do current self- or observer-reported instruments assess? Front Pharmacol. 2020;11:572. doi:10.3389/fphar.2020.00572 32477110 PMC7237632

[4] Taitel M, Fensterheim L, Kirkham H, Sekula R, Duncan I. Medication days’ supply, adherence, wastage, and cost among chronic patients in Medicaid. Medicare Medicaid Res Rev. 2012;2(3):E1-E13. doi:10.5600/mmrr.002.03.A04 24800150 PMC4006393

[5] Rymer JA, Fonseca E, Bhandary DD, Kumar D, Khan ND, Wang TY. Difference in medication adherence between patients prescribed a 30-day versus 90-day supply after acute myocardial infarction. J Am Heart Assoc. 2021;10(1):e016215. doi:10.1161/JAHA.119.016215 33342227 PMC7955468

[6] Rodriguez F, Maron DJ, Knowles JW, Virani SS, Lin S, Heidenreich PA. Association of statin adherence with mortality in patients with atherosclerotic cardiovascular disease. JAMA Cardiol. 2019;4(3):206-213. doi:10.1001/jamacardio.2018.4936 30758506 PMC6439552

