# Supplementary material for: Change in Default Prescription Length and Statin Prescribing Behavior
Source: JAMA Intern Med. 2025 Apr 7;185(6):736–9. doi: 10.1001/jamainternmed.2025.0185 (PMC11976639; doi:10.1001/jamainternmed.2025.0185)
Supplement: Supplement. — Data Sharing Statement [file jamainternmed-e250185-s001.pdf]

Data Sharing Statement

Mehta. Change in Default Prescription Length and Statin Prescribing Behavior. *JAMA Intern Med*. Published April 07, 2025. doi:10.1001/jamainternmed.2025.0185

Data
Data available: No

Additional Information
Explanation for why data not available: Quality improvement study completed with a waiver for informed consent
